# A scoping review of natural language processing of radiology reports in breast cancer

**DOI:** 10.3389/fonc.2023.1160167

**Published:** 2023-04-12

**Authors:** Ashirbani Saha, Levi Burns, Ameya Madhav Kulkarni

**Affiliations:** ^1^Department of Oncology, McMaster University, Hamilton, ON, Canada; ^2^Hamilton Health Sciences and McMaster University, Escarpment Cancer Research Institute, Hamilton, ON, Canada; ^3^Michael G. DeGroote School of Medicine, McMaster University, Hamilton, ON, Canada; ^4^Department of Radiology, McMaster University, Hamilton, ON, Canada

**Keywords:** breast cancer, natural language processing, radiology report, mammography, machine learning, deep learning, artificial intelligence

## Abstract

Various natural language processing (NLP) algorithms have been applied in the literature to analyze radiology reports pertaining to the diagnosis and subsequent care of cancer patients. Applications of this technology include cohort selection for clinical trials, population of large-scale data registries, and quality improvement in radiology workflows including mammography screening. This scoping review is the first to examine such applications in the specific context of breast cancer. Out of 210 identified articles initially, 44 met our inclusion criteria for this review. Extracted data elements included both clinical and technical details of studies that developed or evaluated NLP algorithms applied to free-text radiology reports of breast cancer. Our review illustrates an emphasis on applications in diagnostic and screening processes over treatment or therapeutic applications and describes growth in deep learning and transfer learning approaches in recent years, although rule-based approaches continue to be useful. Furthermore, we observe increased efforts in code and software sharing but not with data sharing.

## 1 Introduction

Female breast cancer is the most commonly diagnosed cancer and is the fifth leading cause of cancer mortality worldwide (1). However, breast cancer survival has improved following advances in systemic therapies (2, 3) and early diagnosis facilitated by mammographic screening (4, 5), especially in countries with a high Human Development Index (HDI) (1). Diagnosis, treatment, and management can be conceptualized as phases along the breast cancer continuum of care (BCCC) with a patient’s entry into the BCCC often occurring with routine mammography for screening (6).

**Figure 1:**
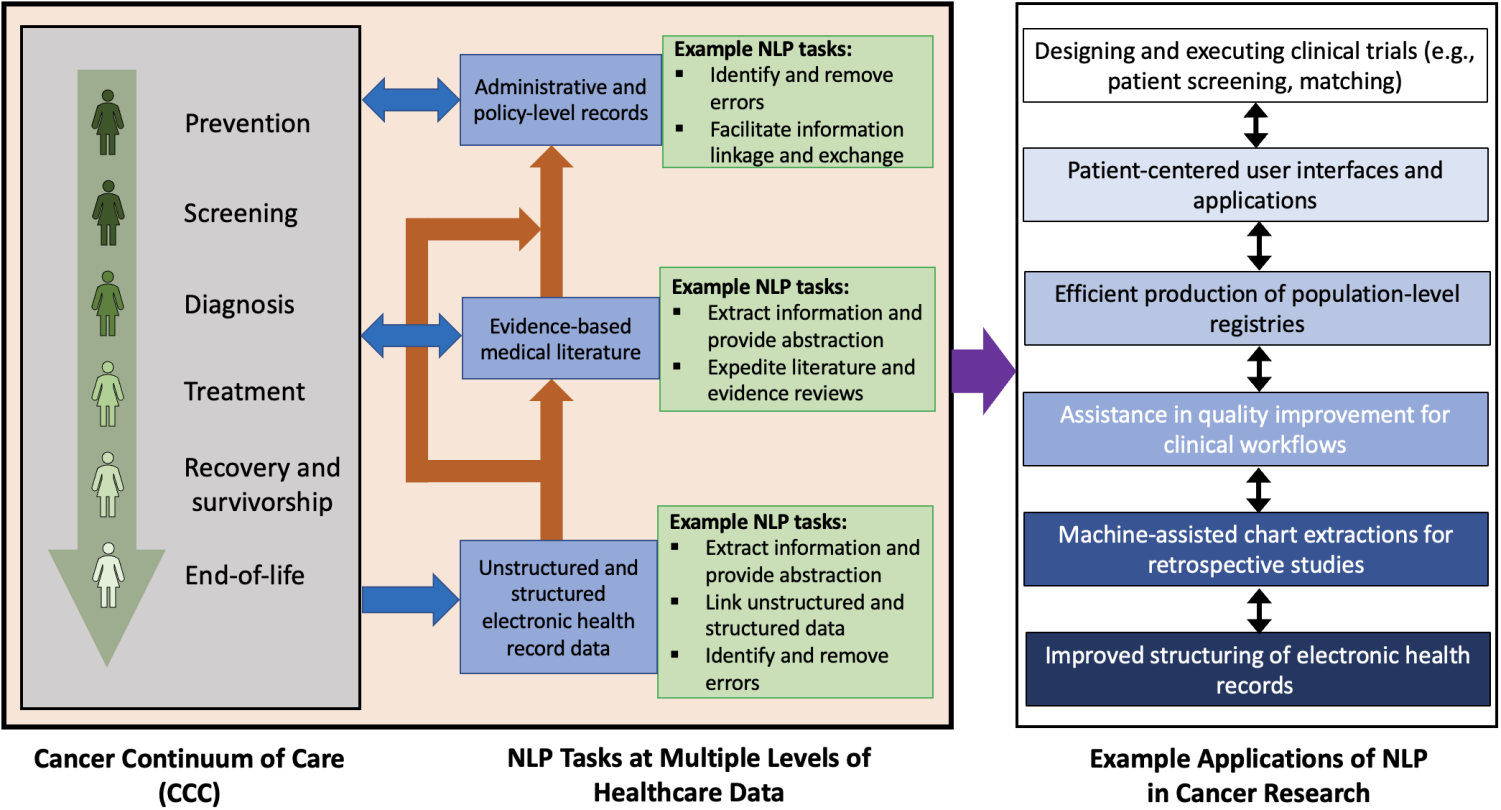
Potential NLP tasks at various levels of electronic healthcare data and some corresponding applications of NLP in oncology. The stages of the cancer continuum of care are as indicated by Cancer Care Ontario (7). The example applications listed may overlap or have mutual influence on each other.

Patients generate data through their interaction with modern healthcare data collection and informatics systems. Improved survival in breast cancer provides more interactions with such systems and results in an increased generation of health records. These health records can be electronic or handwritten and often take the form of unstructured text. Unstructured text can include imaging reports in screening or diagnostic radiology, biopsy reports in pathology, consult and progress notes, surgical reports, discharge summaries, and other written formats that are produced along the BCCC. Large volumes of structured and unstructured text data are produced as byproducts of a patient’s existence in the continuum of care for any cancer. As illustrated in **Figure 1**, this data can leverage natural language processing (NLP) in applications such as clinical trial execution (8, 9), quality improvement (10), population of registries (11), exploration of patterns using text-based data (12), creation of mobile applications for patients (13), and prognostication (14). For example, NLP can be used in clinical trials to search and analyze information in unstructured text, a task that remains difficult to search with simple keyword search. This can be used to improve outcomes or design newer clinical trials altogether. Another use case for NLP can help in quality improvement of dictated medical documents such as clinical notes or radiology reports which can include detection of errors in BI-RADS category, treatment recommendations, or documentation of the side of surgery.

Radiology reports have shown substantial promise to streamline processes and improve healthcare quality (15, 16). In the context of breast cancer, NLP with radiology reports has received particular attention following the implementation of the Breast Imaging and Reporting Data System (BI-RADS) by the American College of Radiology (17). As a starting point for development of NLP systems, BI-RADS provides a convenient search target for rule-based NLP systems. Moreover, the large volumes of electronic text generated across the BCCC, particularly from screening mammography, allows for the creation of datasets of sufficient size and quality for the development of data-driven systems using advanced statistical techniques.

While NLP applications using radiology reports have been reviewed in several studies, there are no published literature reviews on NLP applications of radiology reports that are specific to the management and study of breast cancer. Clinical teams looking to solve specific challenges with NLP are faced with several implementation decisions based on broad range of NLP application domains in breast cancer treatment and research, increasing variety of algorithms that are being developed in parallel in the literature, heterogeneity in data collection and processing, and non-clinical expertise requirements for the successful implementation of a developed NLP system. The goals of this review are to (a) identify areas of interest in the BCCC that are being most addressed by NLP systems in radiology and compare their objectives, (b) discuss the implementation considerations of these NLP systems (e.g., dataset-specific details, NLP task and approach, public availability of data or code, limitations), and (c) share insights to support improvements and research advancements in this interdisciplinary topic.

### 1.1 Overview of current NLP

NLP methodologies constitute a subcategory of artificial intelligence (AI) that build and apply computational models to automate the understanding, representation, and manipulation of human text and speech (18, 19). Unstructured text in medicine contains useful information for clinical and research purposes. It has been established that NLP systems in healthcare settings can extract information from unstructured text data including electronic health records (EHRs) with similar performance to manual extraction by trained professionals depending on the specific extraction task (20–22). In short, NLP has enabled automated and semi-automated processing of unstructured text data at scale.

At a high level, NLP systems can leverage two approaches: either rule-based approaches that rely on human curation of heuristic rules and implementing them to text of interest, or machine learning (ML)-based approaches that independently learn patterns from text data that can be used to perform tasks and produce models. Hybrid approaches with elements from both approaches also exist. Recently, deep learning (DL) approaches have emerged as a subcategory of ML-based NLP systems which include convolutional neural networks (CNNs), recurrent neural networks (RNNs), and related innovations including Bidirectional Encoder Representations from Transformers (BERT). These DL techniques are increasingly being investigated in both non-medical and medical language processing (23–26). The primary limitation of rule-based systems, despite having lower data requirements for development and models that are more interpretable for non-specialists, is that one cannot conceive *a priori* of every possible textual variation, spelling mistake, or alternative phrasing in natural language that refers to a particular medical finding or attribute. ML systems do not require manual rule creation but have greater data requirements for development. DL systems have the greatest requirements in terms of data and specialized technical knowledge, although the development burden has been partially offset by recent advents in transfer learning (27) where powerful pre-trained models, developed with complex architecture and using massive computing infrastructures (e.g., BERT, T5, GPT-3), can be fine-tuned to complete tasks in new domains (28). The subset of ML excluding DL is often referred to as traditional ML or classical ML.

Evaluation of NLP can be completed with several approaches. Holdout validation refers to a model being developed or trained and then deployed on a subset of data reserved or held out for testing after completion of the training process (29). Holdout validation is the simplest type of cross-validation (30). Among other types of cross-validation, *k*-fold cross-validation is commonly used. It splits a dataset into *k* subsets, individually training on (*k*-1) subsets and testing on a final subset until all subsets are tested once, providing an average performance score from all *k* subsets (31). While these approaches are for internal validation, there exist approaches for external validation including independent validation (32) which is testing on a dataset “plausibly related” (33) but independent from the training data in some well-defined aspect (e.g., from other institution, from other time periods, from other disease conditions).

### 1.2 Related work

Pons et al. (34) present the first known systematic review of NLP in radiology, studying 67 articles published up to October 2014. They focused on NLP tasks and grouped the articles into five categories as well as into NLP methodology and tools, limitations and challenges, and future advancements. A pair of review articles build on this work include a literature search through October 2019 by Casey et al. (16) and an assessment on reporting quality of NLP manuscripts in this area by Davidson et al. (35). Luo et al. (36) have an educational article on NLP in radiology that offers clinical use cases and comments on workflow enhancement. Sorin et al. (15) conducted a systematic review for DL-based NLP in radiology.

Among the review articles considering NLP in radiology with an emphasis on cancer, Wang et al. (37) assessed alignment of Minimal Common Oncology Data Elements (mCODE) with NLP-extracted data elements from EHR using articles published between 2010 and September 2020. Hughes et al. (38) discussed the potential of NLP in breast cancer management in their review but focused largely on NLP-assisted literature searches for gene penetrance studies.

## 2 Methods

Our study follows PRISMA guidelines for scoping reviews (39). Searches were performed in PubMed, Web of Science, Embase, and Ovid MEDLINE^®^. The search query required the terms “breast”, “cancer”, and “NLP” or “natural language processing”, as well as one at least one radiological term from the following list: “imaging”, “radiography”, “radiology”, “x-ray”, “mammography”, “mammogram”, “CT”, “MRI”, or “magnetic resonance imaging”.

Following the search queries, included studies must have either developed or evaluated an NLP application using free-text radiology reports for the study of breast cancer. Papers that used multiple data sources were permitted if free-text radiology reports were included among the sources. Similarly, papers that studied multiple diseases were permitted if breast cancer was among the diseases studied. All studies published on or before August 31, 2022 were eligible for inclusion. Manuscripts must have been published in English, but there was no restriction on the language of the radiology datasets. Exclusion criteria included literature reviews, editorial or commentary articles, abstracts for conference poster presentations, and unpublished preprints, including those hosted on archives (e.g., arxiv, biorxiv).

Covidence (www.covidence.org) was used to facilitate the screening process. Screening for inclusion was performed independently by two authors (AS, LB) with disagreements resolved by consensus. A first pass was completed based on title and abstract screening only, followed by a round of full-text screening for inclusion. For all included articles, a reverse snowball search was completed where each citation was considered for inclusion.

Two authors (AS, LB) extracted data elements based on the questions as shown in **Table 1** with conflicting findings resolved by consensus. Several data elements were coded into levels after the extraction of all data to facilitate the presentation of the results. The coding strategies and study-specific code-levels are provided as **Supplementary Material**.

**Table 1:** Questions answered through our study and corresponding data elements.

Number	Question	Data elements
1	When was the study published?	Year
2	Where was the study conducted?	Country
3	What is the title of the study?	Title
4	Where was the study published?	Venue
5	What is the relevance of the NLP system(s) to BC when compared to other cancers/diseases?	BC Relevance
6	In addition to radiology reports, what other data were used?	Other Sources (BC)
7	Which phase from BCCC is most relevant to the study?	BCCC Relevance
8	Were the dataset(s) derived from one or multiple institutions?	Institutions
9	What is the language of the radiology reports?	Language
10	What BC-relevant imaging modalities contributed to the radiology reports?	Imaging Modalities (BC)
11	What BC-relevant radiology procedures contributed to the radiology reports?	Procedures (BC)
12	How many BC-related radiology reports were used in the study?	Reports (BC)
13	How many BC patients were used in the study?	Patients (BC)
14	What type of technical task is being performed by the NLP system?	NLP Task
15	Are some details of the annotation process revealed (e.g., time and effort)?	Annotation
16	Are career-level details of the annotators included?	Expertise
17	Was text pre-processing described?	Text Pre-processing
18	What type of NLP system(s) are used?	NLP Type
19	Did the NLP developed used BERT or its derivatives?	BERT Usage
20	Was the goal related to evaluating an existing system or to both develop and evaluate?	Development/Evaluation
21	How would you describe the evaluation process?	Evaluation Process
22	What is the data granularity at which the tool is evaluated?	Evaluation Level
23	What performance measures are used to evaluate the systems?	Performance Measure
24	Is the data used partly or fully available to researchers?	Data Available
25	Is the codebase used or software developed partly or fully available to researchers?	Code/Software Available
26	What are the commonly stated limitations by the authors?	Limitations

BC, Breast Cancer; BCCC, Breast Cancer Continuum of Care; BERT, Bidirectional Encoder Representations from Transformers; NLP, Natural Language Processing.

## 3 Results

**Figure 2:**
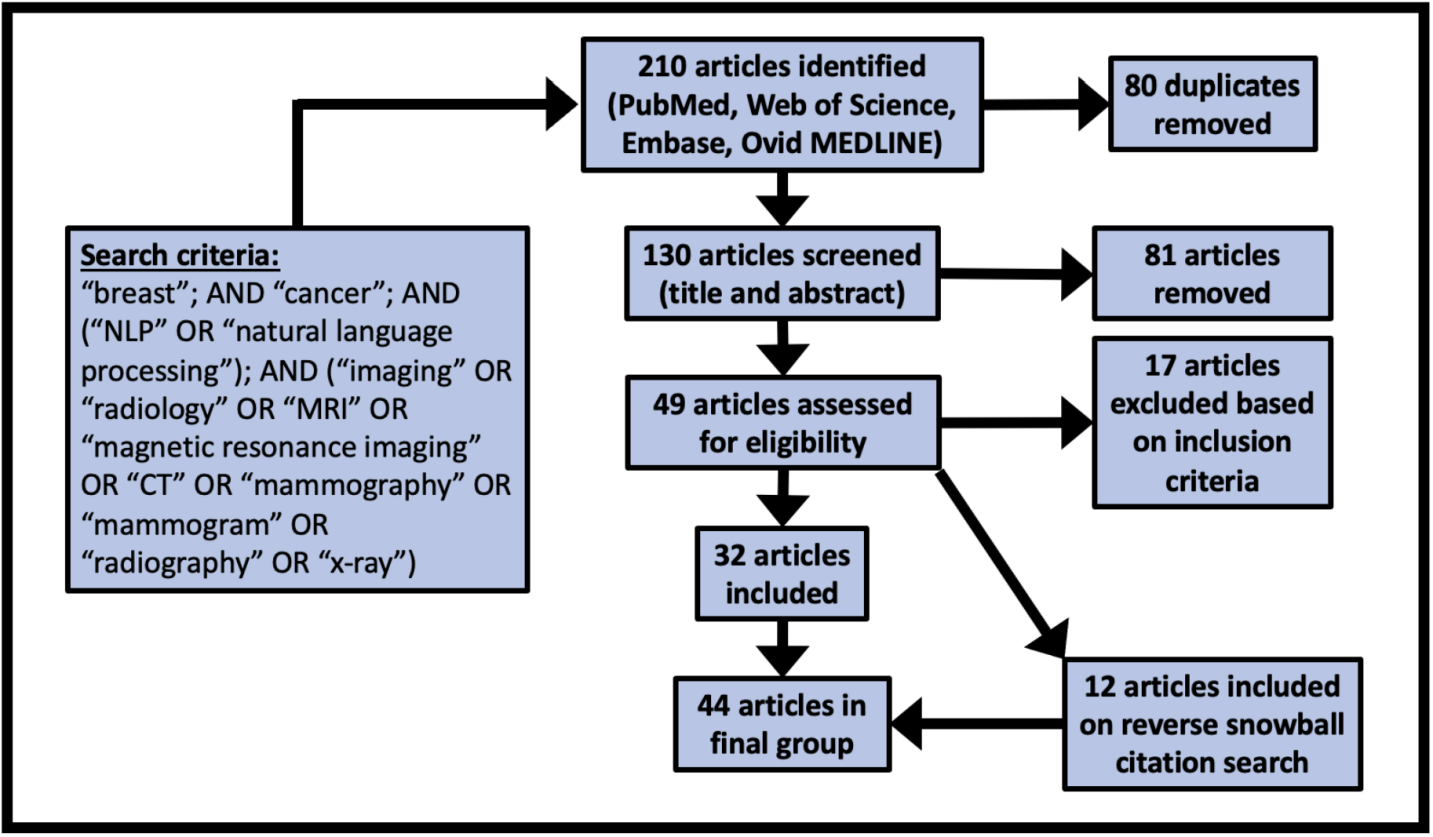
PRISMA diagram demonstrating the search and identification process for the scoping review.

We identified 44 studies suitable for final review. The PRISMA diagram in **Figure 2** demonstrates the stepwise details of the identification process. The 26 data elements as outlined in **Table 1** are available for each of the included 44 articles as **Supplementary Material**. Descriptive statistics on the data elements related to sections 3.1 – 3.4 (see **Table 2**) and for data elements related to sections 3.5 – 3.8 (see **Table 3**).

**Table 2:** Descriptive statistics related to publication timeline, venue, language of radiology reports, relevance to breast cancer and BCCC, and type of data used.

Data Element	N = 44 (%)
Year	
1997 - 2002	2 (4.5%)
2003 - 2009	3 (6.8%)
2010 - 2015	10 (22.7%)
2016 - 2022	29 (65.9%)
Venue	
Conference	7 (15.9%)
Journal	37 (84.0%)
Language	
Chinese	4 (9.1%)
Chinese and English	1 (2.3%)
Dutch	1 (2.3%)
Dutch and English	1 (2.3%)
English	32 (72.7%)
Italian	1 (2.3%)
Persian	1 (2.3%)
Polish	1 (2.3%)
Portuguese	1 (2.3%)
Spanish and English	1 (2.3%)
Country	
Non USA	16 (36.3%)
USA	28 (63.6%)
BCCC Relevance	
Diagnosis	4 (9.1%)
Follow-up	5 (11.4%)
Follow-up/Palliative	2 (4.5%)
Not particular	1 (2.3%)
Screening	5 (11.4%)
Screening/Diagnosis	23 (52.3%)
Screening/Diagnosis/Treatment	1 (2.3%)
Screening/Treatment	1 (2.3%)
Throughout	1 (2.3%)
Treatment to Palliative	1 (2.3%)
BC Relevance	
Across several diseases	3 (6.8%)
BC only	29 (65.9%)
BC only, Applicable to several diseases	3 (6.8%)
Independent application to several cancers	4 (9.1%)
Independent application to several diseases	5 (11.4%)
Other Sources (BC)	
Radiology	34 (77.3%)
Radiology and Clinical Notes	1 (2.3%)
Radiology, Pathology, and Clinical Notes	5 (11.4%)
Radiology, Pathology, Clinical Notes, and Demographics	1 (2.3%)
Radiology, Pathology, Images, Demographics	1 (2.3%)
Various	2 (4.5%)

BC, Breast Cancer; BCCC, Breast Cancer Continuum of Care.

Note that, within each data element the percentages may not add up to exact 100% due to rounding.

**Table 3:** Descriptive statistics related to type of study and NLP usage, source and size of datasets, and model evaluation.

Data Element	N = 44 (%)
Development or Evaluation	
Development and Evaluation	39 (88.6%)
Evaluation	5 (11.4%)
NLP Type	
Classical ML	7 (15.9%)
Classical ML, DL/RNN	3 (6.8%)
DL/RNN	8 (18.2%)
DL/RNN, Hybrid	1 (2.3%)
Hybrid	3 (6.8%)
Rules	18 (40.9%)
Rules, Classical ML	1 (2.3%)
Rules, Classical ML, DL/RNN	2 (4.5%)
Rules, DL/RNN	1 (2.3%)
BERT Usage	
BERT-based	6 (13.6%)
Not BERT-based	38 (86.4%)
Institutions	
Multiple institutions	6 (13.6%)
Single Institution	35 (79.5%)
Single institution + regional cancer registry	2 (4.5%)
Unclear	1 (2.3%)
Radiology Reports (BC)	
<1000	13 (29.5%)
1000 - 10000	8 (18.2%)
10000 - 50000	4 (9.1%)
50000 - 100000	3 (6.8%)
>100000	8 (18.2%)
Unclear	8 (18.2%)
Patients (BC)	
100 - 1000	6 (13.6%)
1000 - 10000	8 (18.2%)
10000 - 50000	1 (2.3%)
50000 - 100000	1 (2.3%)
Unclear	28 (63.6%)
Evaluation Process	
Cross-validation	6 (13.6%)
Cross and Holdout Validation	1 (2.3%)
Holdout and Independent Validation	3 (6.8%)
Holdout Validation	25 (56.8%)
Independent Validation	8 (18.2%)
Unclear	1 (2.3%)

BC, Breast Cancer; DL, Deep Learning; ML, Machine Learning; RNN, Recurrent Neural Networks.

Note that, within each data element the percentages may not add up to exact 100% due to rounding.

### 3.1 Publication timeline, venue, and language of reports

Most studies (24) were published on or after 2018. Twenty-eight papers were from the United States, followed by China (5) and the Netherlands (3). The majority of studies were published in journals (36) and all remaining studies were full conference publications (8). Most studies worked on radiology reports written only in English (31), followed by other languages including Chinese (4), Dutch (1), Italian (1), Persian (1), Polish (1), and Portuguese (1). Three articles addressed NLP procedures using datasets including more than one language such as English and Dutch (1), English and Spanish (1), and English and Chinese (1). One study developed NLP for scanned paper documents in English (40).

### 3.2 Relationship of the studies with the BCCC

#### 3.2.1 Screening and diagnosis

Thirty-five of the 44 articles addressed clinical and technical NLP issues pertaining primarily to the screening and diagnosis of breast cancer or were based on processing text reports focusing on these phases. Of these, 28 studies include radiology reports from mammography (40–67). Primary objectives of these 28 studies included extracting relevant information based on pre-defined terms (42, 50, 56, 59, 62, 63, 65–67), identifying and characterizing abnormal findings (e.g., location, laterality, related sentences) (44, 48, 49, 58, 60), inference of BI-RADS final assessment categories by analyzing the findings section of radiology reports (46, 55), identifying abnormal screening results requiring follow-up or as determined by subsequent pathology reports (40, 41, 43), determination of breast tissue composition class (51), and risk assessment or risk stratification of findings within BI-RADS categories for malignancy (45, 53). Two studies are related to the development of NLP techniques to assist radiologists by providing word suggestions (47) and proposition of new RADLex dictionary terms (64). Validation of pre-existing NLP tools such as BROK for identification of BIRADS final assessment category (54), IBM content analytics software for extracting abnormal mammogram results (57), and MEDLEE and LEXIMER, respectively, for identification of suspicious findings from mammogram reports were carried out (52, 61).

Ultrasound reports were used in six studies (48, 50, 54, 68–70). Extraction of BI-RADS findings (68), association of body locations (69), and automated detection and correction of misspellings (70) were performed in three of these studies that did not specify usage of mammograms. MRI reports relating to breast cancer were also used in six studies (42, 48, 50, 54, 71, 72). Two of these studies focused on extraction of MRI BI-RADS descriptors and categories (71) and identification and related information of index lesions (72). Computed tomography (CT) scans (48, 73) and digital breast tomosynthesis (50) were also used in a few studies.

#### 3.2.2 Treatment, follow-up, and palliative care

The two MRI-related studies (71, 72) are also relevant for the treatment of breast cancer patients. The remaining 9 of 44 studies are more closely related to treatment, follow-up, and palliative care. Three studies used only radiology reports to determine outcomes, sites of metastasis (73, 74), or clinical inflection points (e.g., worsening prognosis, transition to therapies of palliative intent) (75). Six other studies pertaining to follow-up and palliative care used other clinical text notes in addition to radiology reports including progress notes (14, 76–80). Among these six studies, four developed models for breast cancer only (76–78, 80). The remaining two included Morin et al.’s study where independent models for three types of cancer were developed (14) and Banerjee et al.’s study concerning the development of a model providing survival estimates of patients for more than eight types of cancers (79).

The study by Zhang et al. (80) used six types of clinical notes to develop a breast cancer information model that spans across the BCCC for patients who underwent surgery. One study examined the effectiveness of using multiple data sources on top of radiology reports to determine hospital admissions for specific diseases (81). Given the presence of many data sources beyond screening or diagnostic reports, this study was included in our latter category for treatment and follow-up.

### 3.3 Applicability to other cancers or diseases

Fifteen of the studies included data from other cancers or diseases in addition to breast cancer. Of these studies, four developed or evaluated NLP systems using the same methodology as for other cancers (14, 43, 57, 73). Five studies developed or evaluated NLP systems for non-cancer disease or disease sites including diabetes (66), disease observable on bone radiograph (40), disease observable from head and neck, abdominal, or pelvic ultrasounds (70), neuroimaging (69), or various diseases for which confirmation was required by pathology or further radiology studies (81). Finally, six studies developed or evaluated models that apply across various diseases and cancers (61, 74, 75, 78, 79, 82), three of which specifically evaluated model performance using breast cancer data (74, 75, 82).

### 3.4 Radiology reports and other sources of clinical information in the same NLP system

Thirty-four of 44 included studies did not use other sources of text, apart from the BC-related radiology reports, for their individual NLP development and evaluation. Other than radiology reports, usage of texts from pathology reports and different combination of other clinical notes (e.g., oncologists’ notes, progress notes, discharge summaries) were reported by 9 studies (14, 53, 76–82). Image guided biopsy reports (42) and radiology images (53) were also used in the same system using NLP for the tasks.

**Figure 3:**
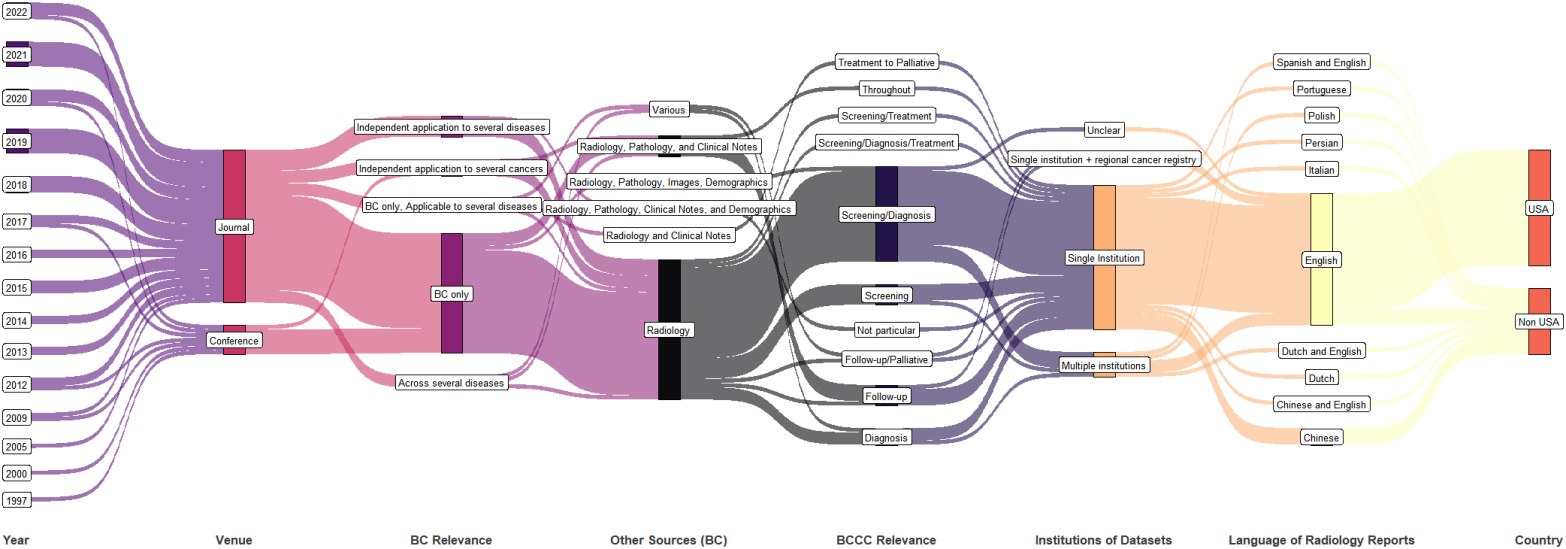
Synthetic analysis (Sankey plot) showing the relationship among publication year, venue, diseases studied, data type, number of institutions in the dataset, radiology report language, and country.

A summary of the year, publication venue format, breast cancer relevance, data type, institutional collaboration in datasets, language of radiology reports, and country of publication is shown in **Figure 3**. The number of studies is growing every year. Though single-institution studies dominate, multi-institution studies are also being conducted (elaborated further in section 3.6 discussing datasets). Multiple data sources (other than radiology reports) are used mostly when the studies apply to post-diagnosis part of BCCC.

### 3.5 NLP tasks and implementations

Twenty-three studies performed information extraction from unstructured text data. Information extraction includes locating relevant terms from the reports, information about an abnormal finding, or structuring the report text into a template of pre-defined fields. Classification was the primary goal of 17 studies for diagnosis, prognostication, medical history, decision support, and cohort formation. Two studies presented methodology for producing optimized risk scores and probability of malignancy. Word suggestion or auto-completion tasks were handled in two studies.

Rule-based NLP approaches were used in 22 studies. Among these, four studies used rule-based and ML-based techniques separately for different AI tasks or for comparison among different algorithms for one task. Classical ML was used in 13 studies. Five of these 13 studies used DL/RNN. In total, 15 studies used DL approaches including the use of RNNs. Hybrid approach of rule-based and ML-based techniques was considered in three studies. Among the studies that used DL, BERT-based approaches were used in 6 studies.

### 3.6 Datasets

Thirty-five manuscripts described working with datasets sourced from a single institution (i.e., hospital, healthcare center, or hospital network). Of the remaining works, six developed or evaluated NLP models with datasets from multiple institutions, two included regional cancer registry data along with data from single institution, and in one study this was not described.

For the 39 studies that performed development and evaluation, the size of datasets in terms of the number of radiology reports varied from less than 1000 to over 100,000. A significant portion of these studies (24/39) did not clearly specify the number of breast cancer patients present in their sample of development or evaluation. Among those that reported, the number of patients remained below 100,000 taking together both development and evaluation. For the five studies that performed evaluation only, the number of radiology reports used was below 10,000 and the number of patients remained close to 1000, though most studies did not report number of patients separately (we assume that one patient may have more than one radiology report present in a given dataset unless stated otherwise).

### 3.7 Annotations

To evaluate NLP models, a human annotator often manually performs the task so that computer performance can be compared to the annotations from the trained clinician or domain expert, called ground truth or reference standard annotations. The type of annotation depends on the type of research question. Annotations can be at the level of entire reports, specific findings described in a report, report sections, or individual sentences, terms, phrases, or words.

Eight studies reported using pre-annotated datasets (e.g., data from an earlier study). Of the remaining studies, 33 described some level of detail about their annotation process. Details included agreement or variability analysis of annotation (12), time estimate of annotation or workload (4), and iterative correction of labels (3). Thirty-three studies reported some information about the expertise of annotators. Annotators included students (both medical and non-medical), professional coders or abstractors, oncologists, and radiologists at various stages of training or experience.

### 3.8 Evaluation processes and metrics

Most studies reported their way of evaluation as cross-validation (6), holdout validation (25), independent validation (8), a combination of cross-validation and holdout validation (1), or a combination of holdout and independent validation (3).

**Figure 4:**
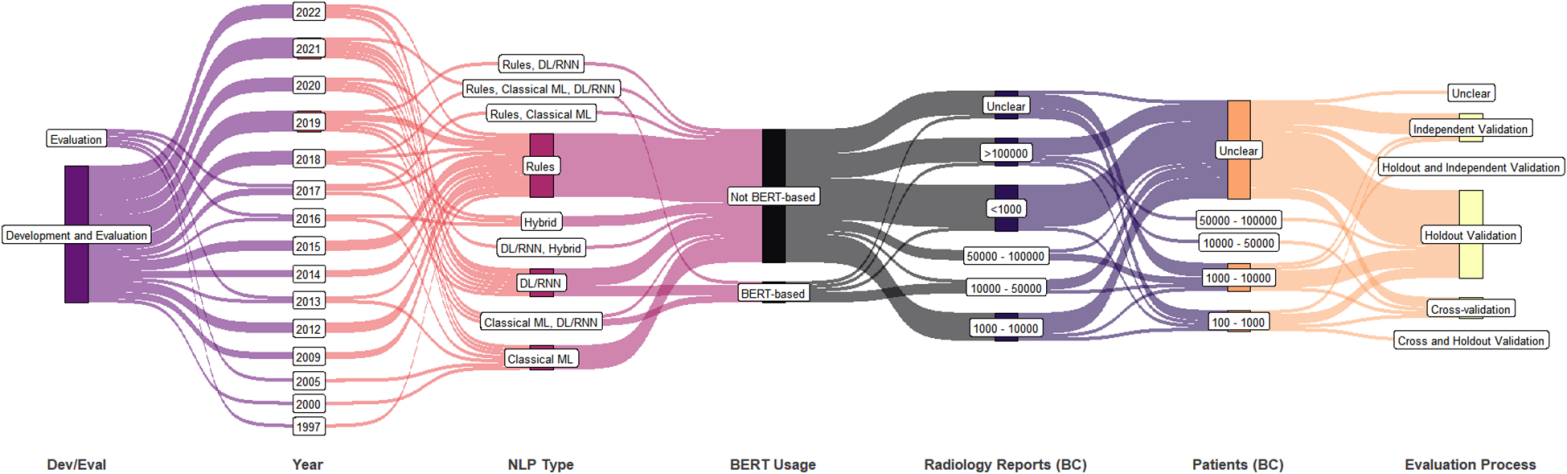
Synthetic analysis (Sankey plot) showing the relationship among NLP development and/or evaluation (Dev/Eval), year of publication, NLP approach, BERT usage, counts of breast cancer patients and radiology reports, and evaluation process. (DL, deep learning; ML, machine learning; RNN, recurrent neural network; BERT, bidirectional encoder representations from transformers).

A summary of further study attributes is shown in **Figure 4** including the technical purpose (i.e., development and/or evaluation), publication year, NLP approach, usage of BERT, number of breast cancer radiology reports, number of breast cancer patients, and the evaluation process. After 2017, most studies performed both development and evaluation. Though ML-based techniques are being used heavily, rule-based techniques are still being actively considered at present. Of the six studies that included over 100,000 breast cancer related radiology reports post 2017, two studies used datasets from multiple institutions and performed independent validation.

Similar to the possible levels of annotation described above, evaluation was performed at the level of patients (10), radiology reports (9), individual findings (1), individual words (2), or a combination of these levels (21).

All studies reported usage of one or more evaluation measures with most studies reporting multiple measures. The most widely used evaluation measures are F1 score, precision, and recall. Other metrics include area under receiver operator characteristic curve (AUC), concordance, Harrell’s c-index, Brier score, execution time, confusion matrices and counts or rates of true positive (TP), true negative (TN), false positive (FP), and false negative (FN) findings. One study designed a metric called radiologist’s process evaluation (RPE) derived from their feedback in implementing a system. Another study reported use of confidence flags, to specify the degree of review needed, on the outcomes produced.

### 3.9 Public availability of data and code or software

Three studies indicated that their datasets were either fully or partially publicly available, two of which leveraged existing publicly available data in their work. In total, 12 studies indicated that their code or software was publicly available and also provided a valid reference for that, including the three studies with available data.

### 3.10 Limitations

Thirty-nine of 44 articles included at least one paragraph, section, or discussion that explicitly stated limitations of their own research methods. Of the five articles that did not, three were conference papers and two were full manuscripts.

While many articles described limitations regarding the technical details of their own specific models and approaches, there were several commonalities in the broad limitations described across the included studies. Twenty-eight articles indicated a possible lack of generalizability or presence of bias to other institutions likely having different templates, abbreviations, practitioners, and referral biases. All but two of these articles used single-institution datasets; one study with multi-institution data stated that further experiments on diverse datasets are required to test generalizability. Twenty studies described other sources of possible sample bias in their training sets, with examples including a lack of normal results or lack of certain BI-RADS categories in the training data or stating that the patient population only includes those with clinical conditions or histories where biopsy was indicated. Six studies drew attention to their small datasets and/or training sample sizes, implying a risk of overfitting in their models. Fifteen studies criticized the veracity and/or quality of their underlying clinical data. These included discussions on radiology reports not always containing all pertinent information (e.g., due to radiologist error or implying that some findings are assumed negative by omission) and concerns that EHR data results in noisier datasets than radiology report datasets alone, and that patients may have information stored in other hospital networks or simply not have all encounters recorded in sufficient detail with regards to cancer metastasis or recurrence. Four studies mentioned relying on pre-labelled data (e.g., from prior research, ICD codes, or dictionary) or on pre-defined radiology report headings that were specific to their institution.

In 9 studies, some limitations were specific to the rule-based approach in the paper. Three studies described that despite the best efforts of research and clinical teams, a pre-defined set of rules cannot account for every conceivable clinical finding and/or linguistic description of a given clinical finding, including stylistic variations or spelling mistakes as examples. Three other articles describe the concept of model drift and/or domain shift, where a real deployment of even a theoretical perfect rule-based system would require that rules be updated on a regular basis post-deployment in the face of new institution protocols, staff, or clinical findings. We note that while these issues were only described for rule-based studies in these articles, ML-based methods are also vulnerable to these issues. Thirteen studies discussed limitations in the technical implementation related to pre-training, fairness, poor performance for complex scenarios, imperfections in pre-processing or earlier stages of implementation, data imbalance and imperfections.

## 4 Discussion

We performed a scoping review of research articles using NLP with breast cancer radiology reports. For this review, we included articles from several databases, did not have a start date for the works we included, and included both journal articles and full-text papers from conference proceedings. We extracted 26 types of information from 44 included studies and summarized our findings to understand this cross-disciplinary field in terms of current state-of-the-art techniques and future opportunities that may arise from gaps in literature.

While most radiology reports were from mammography studies, CT, MRI, ultrasound, and digital breast tomosynthesis were also present. All but one paper had radiology reports restricted only to imaging, while one paper also included reports from other radiology procedures. Apart from one study that worked on scanned documents, most studies focus on electronic text reports.

Most studies address breast cancer during the screening and/or diagnostic phases of the BCCC. Many studies focus on information extraction from radiology reports to structure existing information. Though there is no consensus or standard on the structured information that can be derived in this manner, some findings often targeted by studies include final BI-RADS assessment category as well as abnormal findings and their descriptors. Structured information can be used to populate registries, perform quality assurance, assist in cohort selection, and facilitate large-scale data gathering. Most studies used radiology reports as the only source of data and were based on single-institution datasets.

Relatively few studies are related to other BCCC phases. Most of these studies use pathology notes or various other clinical notes for NLP in addition to the radiology report. The models can also be designed to include non-text data (e.g., structured demographic data or radiology images). All of these studies used single-institution data, potentially indicating an increased challenge of curating datasets including different types of clinical notes and from different sources from multiple institutions for post-diagnosis phases of the BCCC.

Studies from the United States conducted their work using English-language radiology reports. Elsewhere, English-language radiology reports were most common but present in less than half of these studies, with radiology reports in Chinese, Dutch, Persian, Polish, and Portuguese appearing as well. Of the 12 studies studying radiology reports in a language other than English, 75% were published in or after 2018. This does not come as a surprise, as presence of EHR systems and its language facilitates development/evaluation of NLP. Furthermore, we note that 7 of these 12 studies used classical ML and DL in their work, with two using BERT, indicating an uptick in the most advanced computational techniques available.

In recent years, the usage of DL has increased, and BERT was released by Google for transfer learning applications in 2018, towards the end of the study period of the prior literature reviews of NLP in radiology. Of the 15 included articles that use DL, only three were published in 2017 or 2018 with the rest published between 2019 and 2022. Six articles used BERT. While all 15 DL studies performed model development (rather than evaluation alone), only six used datasets containing 10,000 or more radiology reports. Nevertheless, rule-based techniques, classical ML, and hybrid techniques continue to be developed in the literature through 2022. Despite the increase in usage of data-driven DL and ML techniques, availability of public datasets is rare. Several studies described annotation procedures in their model development that often depended on rare and cost-intensive expertise, but such datasets are not released for re-use or external evaluation. In comparison, more studies are releasing code accompanying their work, as 7 (50%) of the studies published since 2020 have released their code in comparison to 5 (17%) of the studies prior to 2020. Increased availability of code reflects a trend towards transparency and assists other groups in assessing the reproducibility of results in other contexts.

Generalizability and bias were the most stated limitations of the works, apart from the limitations caused by small sample set and data quality issues. In this scoping review, the earliest paper was published in 1997 with many publications in the ‘00s, although modern volumes of electronic data for analysis have only become widespread more recently with the rise of EHR systems, and they are rarely publicly available. This is understandable in the context of patient confidentiality and data privacy laws. Given that the development an NLP model from scratch is a resource-intensive endeavour, both in terms of dataset collection and algorithm implementation and/or evaluation, having public datasets available would allow teams worldwide to focus on the algorithmic development piece of applying NLP to radiology reports, conduct independent validation, and build more robust models. Efforts in this direction, to enable collaboration across institutional boundaries and to avoid needlessly repeating dataset curation for algorithm development, are necessary to address the current limitations of generalizability and broader implementation of NLP models.

Our scoping review should be interpreted in the context of its limitations. Our search could have included more papers with the usage of more search query terms, including additional optional query terms for other imaging modalities. While our search criteria explicitly included imaging modalities that are most common for screening and staging of breast cancer, it also included several radiological terms that are agnostic to a particular modality, and our final collection of studies reflects the full range of medical imaging relevant to the BCCC. Moreover, we performed reverse snowballing and included more studies through this process spanning several imaging modalities including those not explicitly stated within our search query terms. Another limitation is that we performed categorization after the data extraction was complete. Thus, our categories might have bias based on the articles included. Several fields were categorized based on relevance to breast cancer and may not necessarily be the same categorizations if another disease was studied. We did not perform a critical analysis of the studies included due to the broad diversity of applications, NLP tasks, data sources, and languages studied.

## 5 Conclusion

Automated processing of radiology reports has significant impact on different phases of the BCCC, and the diagnosis and screening phases received the majority of research attention. The applications of NLP can automate mundane tasks to allow clinicians to focus on other complex cases or tasks, allow for epidemiological retrospective research of breast cancer, and allow for widespread quality control measures for routine mammography and breast cancer treatment. The field is growing in terms of publications per year and usage of advanced text-processing AI tools such as BERT for transfer learning and better performance. Expanding the generalizability and reduction of bias are important for the increasing the applicability of the NLP tools and to increase the likelihood of eventual widespread adoption beyond a single institution. Though code and software sharing has improved over the years, sharing of datasets can facilitate improving the methodology of future studies.

## Data availability statement

The original contributions presented in the study are included in the article/**Supplementary Material**. Further inquiries can be directed to the corresponding author.

## Author contributions

AS, LB contributed to conception and design of study, performed abstractions from articles, performed the analyses, and contributed to writing the initial draft of the manuscript. LB performed the literature search. AK critically reviewed for clinical applicability and provided radiologist input to the study. All authors contributed to the article and approved the submitted version.
